# Dr. C. C. F. Gay

**Published:** 1872-02

**Authors:** 


### DR. C. C. F. GAY,

One of our Corresponding Editors furnishes us, in this issue, a valuable and interesting article on Strangulated Hernia. Dr. Gay advances some new and important ideas, in relation to the treatment of Strangulated Hernia, which will command the attention of the profession.

We hope our Corresponding Editors, *generally*, will follow in the footsteps of our brother of Buffalo, New York, and send us communications.

